# Methoprene-Tolerant (Met) Acts as Methyl Farnesoate Receptor to Regulate Larva Metamorphosis in Mud Crab, *Scylla paramamosain*

**DOI:** 10.3390/ijms252312746

**Published:** 2024-11-27

**Authors:** Ming Zhao, Wei Wang, Xin Jin, Zhiqiang Liu, Minghao Luo, Yin Fu, Tianyong Zhan, Keyi Ma, Fengying Zhang, Lingbo Ma

**Affiliations:** 1 Key Laboratory of East China Sea Fishery Resources Exploitation, Ministry of Agriculture, East China Sea Fisheries Research Institute, Chinese Academy of Fishery Sciences, 300 Jungong Road, Shanghai 200090, China; zhaom@ecsf.ac.cn (M.Z.);; 2 College of Fisheries and Life Science, Shanghai Ocean University, 999 Huchenghuan Road, Shanghai 201306, China

**Keywords:** methyl farnesoate, *methoprene-tolerant*, metamorphosis, signal transduction, *Scylla paramamosain*

## Abstract

The conserved role of juvenile hormone (JH) signals in preventing larvae from precocious metamorphosis has been confirmed in insects. Crustaceans have different metamorphosis types from insects; we previously proved that methyl farnesoate (MF) can prohibit larvae metamorphosis in mud crabs, but the molecular signal of this process still needs to be elucidated. In this study, *methoprene-tolerant* (*Met*) of *Scylla paramamosain* was obtained and characterized, which we named *Sp-Met*. *Sp-Met* contains a 3360 bp ORF that encodes 1119 amino acids; the predicted protein sequences of Sp-Met include one bHLH, two PAS domains, one PAC domain, and several long unusual Gln repeats at the C-terminal. AlphaFold2 was used to predict the 3D structure of Sp-Met and the JH binding domain of Met. Furthermore, the binding properties between Sp-Met and MF were analyzed using CD-DOCK2, revealing a putative high affinity between the receptor and ligand. In silico site-directed mutagenesis suggested that insect Mets may have evolved to exhibit a higher affinity for both MF or JH III compared to the Mets of crustaceans. In addition, we found that the expression of *Sp-Met* was significantly higher in female reproductive tissues than in males but lower in most of the other examined tissues. During larval development, the expression variation in *Sp-Met* and *Sp-Kr-h1* was consistent with the immersion effect of MF. The most interesting finding is that knockdown of *Sp-Met* blocked the inhibitory effect of MF on metamorphosis in the fifth zoea stage and induced pre-metamorphosis phenotypes in the fourth zoea stage. The knockdown of *Sp-Met* significantly reduced the expression of *Sp-Kr-h1* and two ecdysone signaling genes, *Sp-EcR* and *Sp-E93*. However, only the reduction in *Sp-Kr-h1* could be rescued by MF treatment. In summary, this study provides the first evidence that MF inhibits crustacean larval metamorphosis through Met and that the MF-Met→*Kr-h1* signal pathway is conserved in mud crabs. Additionally, the crosstalk between MF and ecdysteroid signaling may have evolved differently in mud crabs compared to insects.

## 1. Introduction

The mud crab *Scylla paramamosain* is an economically important fishery species in Asian countries. *S. paramamosain* is also one of the most important aquaculture species, with an annual output of 150,000 tons in China. The life cycle of mud crabs includes the fertilized egg and embryonic stages, five zoea stages, one megalopa (M) stage, the first juvenile crab (C1) stage, and then more than ten molting cycles, after which the crab reaches the adult stage. During the entire life of the mud crab, metamorphosis zoea five (Z5) to M stage and M to C1 stage are two key points in the artificial seed breeding of the mud crab, which usually decide the success or failure of one breeding process. Therefore, the metamorphosis regulation mechanism deserves deep exploration. Recently, Ardavan et al. [1] reviewed the molecular mechanism of embryonic development in decapod crustaceans and discussed the pivotal role of signaling pathways such as Hedgehog, Wnt, Notch, MAPK, TGF-β, Jak-STAT, VEGF, and ecdysteroids in the regulation of embryogenesis processes, including egg activation, the maternal-to-zygotic transition, mesoderm development, segmentation, nervous system development, sex determination, germline development, and exoskeleton formation. Both embryogenesis and larval metamorphosis possess significant morphology changes, as suggested by some similar regulatory mechanisms behind them. However, the most obvious differences between them are that the larvae use an exogenous diet as the developmental energy source while the embryo uses the endogenous vitelline as the energy source, suggesting that there may also be some differences.

Since there are no model animals in crustaceans, the molecular mechanism studies of crustaceans are mainly referring to insects. However, crustaceans and insects possess different metamorphosis types. Take the metamorphosis process of the mud crab as an example; from the Z5 to M stage or M to C1 stage, the larval behavior changes thoroughly: the zoea stage is planktonic with a weak swimming ability, and the M stage has an active swimming ability and generates a pair of chelipeds, while from the C1 stage, the crab folds a contracted tail and becomes a crab type, then starts benthic life. In summary, the metamorphosis of the crab larvae is the transition from early larvae to the juvenile crab stage, the purpose of which is mainly to prepare for growth [2]. However, in holometabolic insects, the metamorphosis from larva to pupa and then to the imago (adult) is mainly to prepare for reproduction. Whether these two different metamorphosis types share a similar mechanism needs to be elucidated.

It has been confirmed that the metamorphosis of insects is mainly controlled by ecdysteroids (Ecds) and juvenile hormones (JHs) [3,4]. Metamorphosis is induced by Ecds, while JHs antagonize with Ecds to decide when to metamorphosize. MF is the primary JH in crustacean species, which is the non-epoxidized form or precursor of JH III, the most common type of juvenile hormone (JH) in insects [5]. Previously, both we and other crustacean researchers have confirmed that methyl farnesoate (MF) can inhibit crustacean larval metamorphosis [2,6,7,8], suggesting that there may be some similarities. However, studies in two *Daphnia* species suggested that there may also be some differences. It was found that the insects’ JH receptor methoprene-tolerant (Met) homolog in *Daphnia* could also bind to MF and possessed a higher affinity to MF than to JH III [9], while the JH primary responsive gene *Krüppel homolog 1* (*Kr-h1*) of *Daphnia pulex* lost JH responsiveness [10]. The interesting thing is that *Kr-h1* in two decapod species might still have MF responsiveness, and this responsiveness was only found in the hepatopancreas but not in the ovary [11,12]. Since *Daphnia* species do not undergo metamorphosis throughout their entire life cycle, the role of Met in transducing MF signals to regulate metamorphosis and other development processes in crustaceans remains unclear. Direct evidence to support this mechanism is still lacking.

During insect metamorphosis, ecdysteroids, mainly 20-hydroxyecdysone (20E), trigger the process through the ecdysone receptor–ultraspiracle (EcR-USP) receptor complex, which induces several transcription factors, including *E93* and *Br-C*. E93 then activates autophagy and apoptosis pathways, leading to the degradation of the larval tissues, while Br-C activates the formation of the pupal tissues [13]. Meanwhile, during the larval molt processes, the JH-Met complex recruits cofactors Taiman/steroid receptor co-activator (SRC)/βFtz-F1-interacting steroid receptor co-activator (FISC) to form a transcript complex [14,15]; with the aid of chaperone Hsp83 [16] and nucleoporin Nup358 [17], the complex enters the nucleus and is located at the DNA binding region in the promotor of a key transcription factor *Krüppel homolog 1* (*Kr-h1*) to induce its transcription [11,18]. Then, Kr-h1 inhibited *Br-C* and *E93* expression by binding to their promotor region, further antagonizing 20E’s role in metamorphosis [19,20,21,22]. In addition, JH also induces *Kr-h1* expression in the prothoracic gland (PG), an organ producing the 20E’s precursor ɑ-ecdysone. By reducing both steroidogenesis autoregulation and PG size, high levels of Kr-h1 in the PG inhibit ecdysteroid biosynthesis, thus maintaining juvenile status [23]. The prerequisite for the discovery of various findings in JH’s function is the establishment of Met as the JH’s receptor [24,25,26]. Therefore, the overall goal of this study is to examine the possibility of Met serving as the MF receptor to regulate larval metamorphosis in mud crabs, and also the possible signal pathway of MF, with the hope of advancing molecular mechanism studies of MF.

## 2. Results

### 2.1. Characters of Sp-Met Sequence

**Figure 1:**
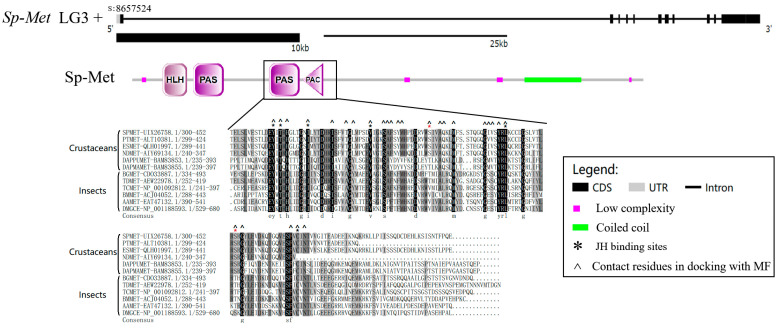
Characters of Sp-Met. Top-down gene structure of *Sp-Met*; protein domain of Sp-Met; alignment of PAS-PAC sequences from six crustaceans and six insects. The * indicates residues that are essential for JH binding [27], and the two variable sites are marked in red.

The mRNA sequence of Met, termed *Sp-Met*, was obtained and deposited in GenBank under the accession number MT597057. The obtained cDNA length of *Sp-Met* is 3677 bp, with a 3360 bp predicted using an open reading frame (ORF), which encodes 1119 amino acids. The putative Sp-Met protein has a molecular weight (M.W.) of 124.666 kDa and an isoelectric point of 6.25. *Sp-Met* is located at the 8,657,524 position of the positive chain at pseudochromosome 3 (LG3), with 11 exons separated by 10 introns. The length of *Sp-Met* is more than 82,341 bp, which is mainly due to the unusually long intron of the first one, with a length of 66,355 bp (Figure 1).

The putative protein sequence of Sp-Met contains one bHLH domain, two PAS domains, and one PAC domain. Sp-Met shows high identity with other crustaceans but relatively low identity with insects. For example, the identity between Sp-Met and Mets of *Portunus trituberculatus* and *Eriocheir sinensis* is 86.90% and 60.11%, respectively, while it is 36.2% between Sp-Met and *Tribolium castaneum* Met. In addition, a 112 peptide chain with 103 glutamine residues and only nine other amino acids are found at the 870–981 position. The unusual numbers of glutamine residues are also found in the Met of *E. sinensis*, but the reason for this remains a mystery.

Since the PAS-b domain of Met has been proven to bind to JHs in other species [9,28], the PAC domain is proposed to contribute to the PAS domain fold [29]. The alignment of PAS-b-PAC and its flank region of Mets from four Decapods, two Cladoceras, and six insects were conducted. The results indicate that six of the eight JH binding sites [28] in the PAS-b domain are conserved in crustaceans and insects, but the other two are not conserved (Figure 1). The fifth residue (Ser357 in *S. paramamosain*) is conserved in these decapod species but is different from *Daphnia* and insects. However, this residue in decapod (Ser) or *Daphnia* species (Thr) is polar and contains basic amino acids, while in insects, it (Val) is non-polar and contains aliphatic amino acids.

### 2.2. The 3D Structure of Sp-Met and Binding Analysis with JHs

**Figure 2:**
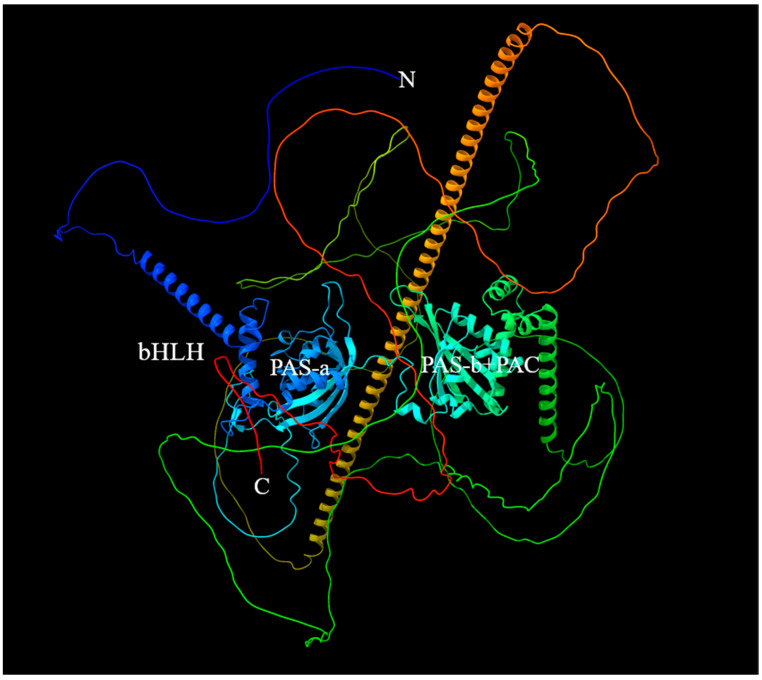
The 3D structure of Sp-Met. Predicted by AlphaFold2.

The 3D structure of Sp-Met was predicted using AlphaFold2 (Figure 2). The results indicate that the bHLH, PAS-a, PAS-b, and PAC domains have a relatively stable structure, but other parts of Sp-Met are relatively volatile, which is indicated by low pIDDT scores (Supplementary Figure S1). Then, the 3D structure of PAS-b-PAC and its flank region (abbreviated as PPF region in the following parts) was also predicted using AlphaFold2. The results suggest that the core structure of the PPF region is stable, as indicated by a high pIDDT score.

**Figure 3:**
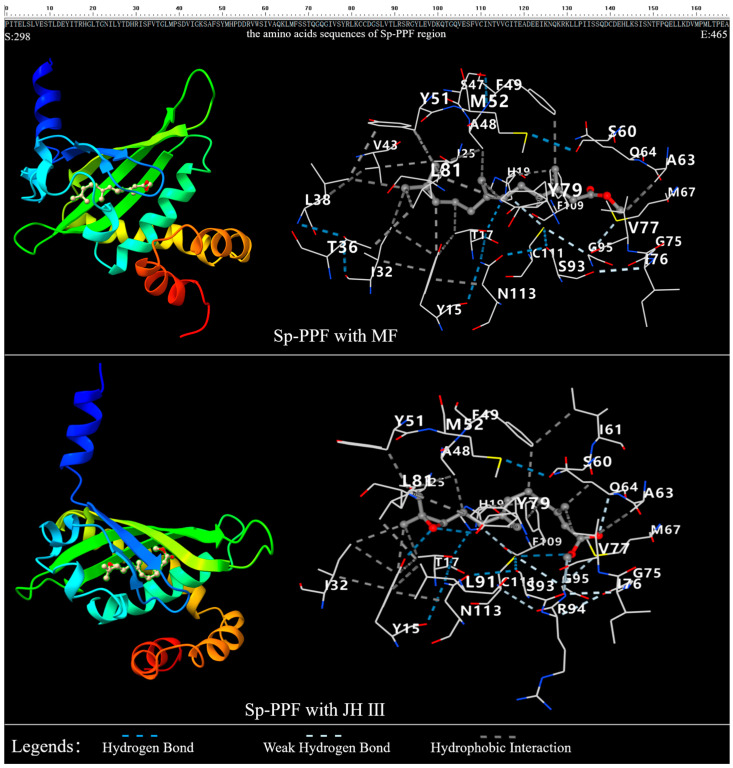
Docking model of Sp-Met (PPF)-MF or JH III and the contact residues. The top sequence is the PPF region of Sp-Met; the left model is the docking model of Sp-PPF-MF or Sp-PPF-JH III; the right model shows the contact residues between Sp-Met and MF or JH III, and the number is the position in the top sequence.

Furthermore, docking between the Sp-PPF and MF or JH III was conducted (Figure 3). The results suggest that high bindings may exist between Sp-PPF and MF or JH III, which are indicated by the −7.2 vina sore (kcal/mol) between Sp-PPF and MF or JH III. Twenty-seven residues docking with MF were identified. Three of the twenty-seven residues in docking with MF, including T333, L335, and S357, are conserved in these decapod crustaceans but are different from *Daphnia* or insect species; Y348, Q361, and V374 are conserved in these decapod crustaceans but are different from other insect species (Figure 1). The contact residues number is 26 when docking with JH III, and T333, L335, V340, and S344 are excluded, while I358, L388, and R391 are included when comparing with the contact residues of MF. Among these residues, Y312 and Y376 are predicted to bind with the oxygen atom of the epoxy group in JH III through the H-bond, while Y348, Y376, and L378 are predicted to contact the CH3 group of the second position of MF through hydrophobic contact.

To analyze the crucial roles of these residues in JH binding, T333A, L335Y, Y348F, S357V, Q361L, and V374S of the Sp-PPF sequences were created and the 3D structures were predicted (Supplementary File S1). Meanwhile, docking analysis between these six PPFs and MF or JH III was conducted. The results suggest that all six replacements increase the binding affinity with JH III, indicated by the high vina score of −8.1 to −7.5; T333A, L335Y, Q361L, and V374S increase the binding affinity with MF, indicated by the high vina score of −7.7 to −7.3, while Y348F slightly decreases the binding affinity with MF (−7.0 of the vina score), and S357V has no obvious effect for the MF binding (Table 1).

### 2.3. Expression Profile of Sp-Met in Adult Male and Female Crabs

**Figure 4:**
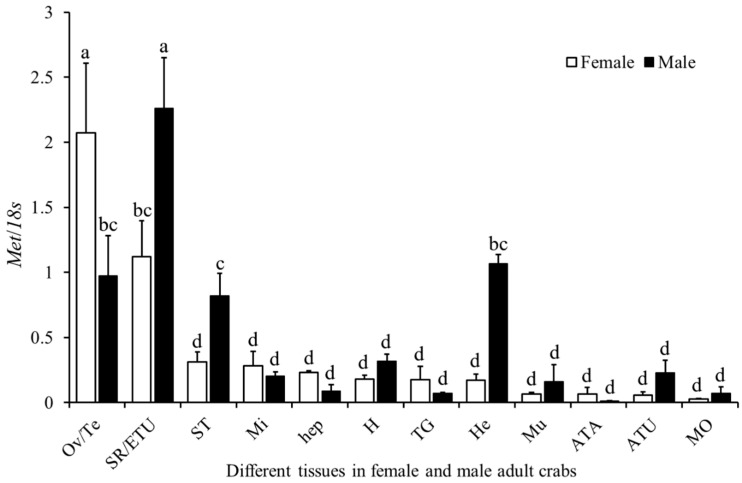
Expression profile of *Sp-Met* in different tissues of female and male adult crabs. The X-axis indicates different tissues, Ov: ovary; Te: testis; SR: seminal receptacle; ETU: ejaculatory duct; ST: stomach; Mi: midgut; Hep: hepatopancreas; H: heart; TG: thoracic ganglia; He: hemolymph; Mu: muscle; ATA: antenna; ATU: antennule; MO: mandibular organ. The Y-axis indicates relative mRNA expression of *Sp-Met*. Different letters indicate significant differences (*p* < 0.05).

The expression of *Sp-Met* in different tissues of adult male and female crabs was examined. The results indicate that significantly different expressions are found between different tissues of the same gender and between the same or related tissues of different genders. In females, *Sp-Met* has the highest expression in the ovary, followed by the seminal receptacle, and low expression is found in other tissues. In males, *Sp-Met* has the highest expression in the ejaculatory duct, followed by the hemolymph, testis, and stomach. The interesting thing is that *Sp-Met* has significantly higher expression levels in the ovary than in the testis but significantly lower expression levels in the seminal receptacle than in the ejaculatory duct. Also, significantly lower expression levels were found in the hemolymph and stomach of females compared to males. In addition, *Sp-Met* also has a slightly higher expression in the midgut, hepatopancreas, thoracic ganglia, and antennae of females than those in the males, but has a slightly lower expression in the heart, muscle, antennule, and mandibular organ of females than those in males, although these differences were not significant (Figure 4).

### 2.4. Spatial Expression of Sp-Met and Sp-Kr-h1 During the Larval Development

**Figure 5:**
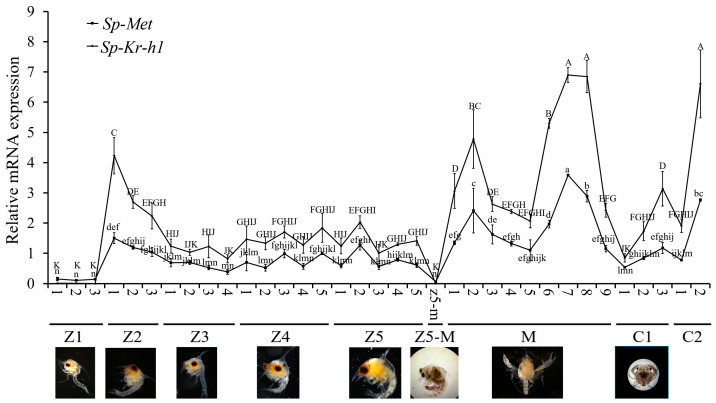
Spatial expression profile of *Sp-Met* and *Sp-Kr-h1* during the larva development. The X-axis represents the days corresponding to different larval stages, with “Z + number” denoting various zoea stages, “M” indicating the megalope stage, and “Z5-M” signifying the metamorphosis from Z5 to M. “C + number” refers to different crab stages. The Y-axis illustrates the relative mRNA expression levels of *Sp-Met* and *Sp-Kr-h1*. Distinct lowercase and uppercase letters indicate significant differences in the expression levels of *Sp-Met* and *Sp-Kr-h1*, respectively (*p* < 0.05).

During larval development, the expression of both *Sp-Met* and *Sp-Kr-h1* are low during the Z1 stages, increase significantly on the first day of Z2, decrease gradually until the last day of Z3, fluctuate during the Z4 and one to four or five days of Z5 stages, and then decrease until rarely detected at the Z5 to M metamorphosis. During megalopa development, the expression of *Sp-Met* and *Sp-Kr-h1* reaches two peaks on the second or seventh day, with one dip on the fifth day, and then decreases gradually from the seventh day until the megalopa metamorphosis. When reaching the first juvenile stages (C1), the expression of *Sp-Met* and *Sp-Kr-h1* increases gradually from the first day to the last day; after decreasing slightly on the first day of C2, the expression of these two genes increases again (Figure 5). The correlation coefficient between the expression patterns of these two genes is 0.97 (*p* < 0.00001), suggesting a highly correlated relationship between these two genes.

### 2.5. Function Studies of Sp-Met During Larval Metamorphosis

**Figure 6:**
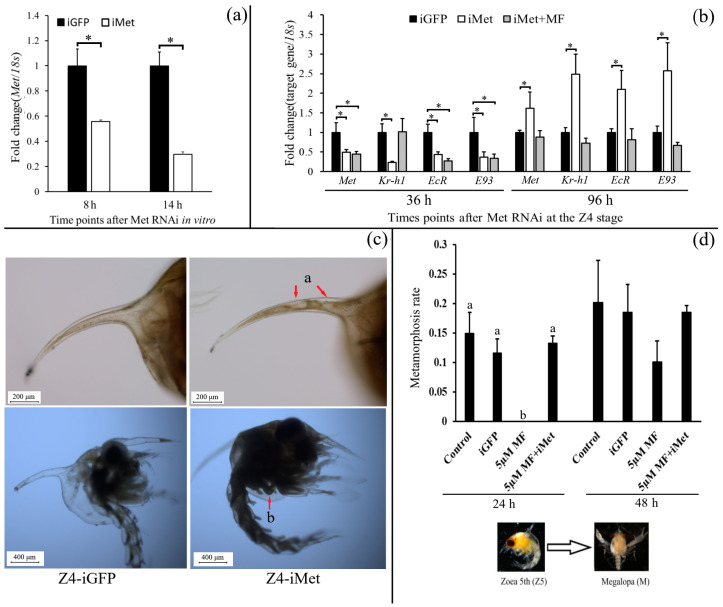
Function study of *Sp-Met* during larva development. (**a**) Examination of RNAi efficiency on *Sp-Met* using the in vitro hepatopancreas culture. (**b**) The expression of *Sp-Met*, *Sp-Kr-h1*, *Sp-EcR*, and *Sp-E93* after RNAi and MF rescue at zoea 4th stage. (**c**) The phenotype of zoea 4th larva after *Met* knockdown, the letter a and red arrow indicate the degradation of the tissues in the spike at the carapace, and the letter b and the red arrow indicate the newborn chela bud; (**d**). Knockdown of *Sp-Met* rescues the prohibitory effect on Z5 to M metamorphosis. Different letters or * indicate significant differences (*p* < 0.05).

In vitro, a hepatopancreas culture was used to examine the RNAi efficiency on *Sp-Met*. The results indicate that the expression of *Sp-Met* decreased by about 45% and 71% in the iMet group at 8 h and 14 h, respectively (Figure 6a). In the in vivo experiment, the expression of *Sp-Met*, *Sp-Kr-h1*, *Sp-EcR*, and *Sp-E93* was reduced by about 51%, 78%, 57%, and 63%, respectively, when compared with the iGFP group at 36 h after *Met* siRNA was added. The addition of MF at 12 h can rescue *Sp-Kr-h1* reduction to the control level, but not *Sp-Met*, *Sp-EcR*, and *Sp-E93*. After 96 h, the expression of *Sp-Met* in the iMet group increased and was significantly higher than that in the iGFP group, suggesting that the siRNA might be degraded (Figure 6b). Meanwhile, the expression of *Sp-Kr-h1*, *Sp-EcR*, and *Sp-E93* is also increased in the iMet group compared with the iGFP group.

On the first day of Z4, the knockdown of *Sp-Met* causes some precocious metamorphosis phenotypes in about 75% of individuals, including the degradation of the tissues in the spike at the carapace and the newborn chela bud (Figure 6c). Since we previously proved that MF can prohibit Z5 metamorphosis, the knockdown of *Sp-Met* was also conducted at the Z5 stage to investigate whether the prohibitory effect of MF is transduced by *Met*. Until 24 h after the experiment started, 5 µM MF completely inhibited Z5 metamorphosis, while the metamorphosis rate of the 5 µM MF + iMet group has no significance compared with the control group, suggesting that the inhibitory effect of MF during Z5 metamorphosis can be rescued by the knockdown of *Met*, indicating that the inhibitory role of MF on Z5 metamorphosis may be transduced by *Sp-Met* (Figure 6d).

## 3. Discussion

The sesquiterpenoid juvenile hormones play a critical role in various development processes of arthropod species, including metamorphosis, reproduction, diapause, cast differentiation, behavior, immunity, etc. [13,30,31]. Over the past decades, the most intriguing finding was the establishment of Met as the JH receptor in insects [19,24]. After that, many interesting findings have been made in insects’ endocrinology [32,33,34,35]. In this study, the function of *Met* in the economic species *Scylla paramamosain* was characterized and investigated.

Sp-Met possesses a conserved bHLH and two PAS domains, and the unusual thing is that a 112 peptide chain with 103 glutamine residues is found in the C-terminal of Sp-Met, and a similar thing was also found in the Met of *E. sinensis* [36]. A possible explanation is that these peptide sequences might function during the interaction with other nuclear receptors since a highly elongated and asymmetric conformation is also found in Germ cell-expressed protein (GCE) of the *Drosophila melanogaster* [37], which is crucial for the interaction interface with Fushi Tarazu factor-1 (FTZ-F 1). In addition, since crustaceans mainly use methyl farnesoate (MF) as the juvenile hormone [5], the JH bind domain should be different in insects and crustaceans. It has been proven that Met of *Daphnia* can interact with steroid receptor co-activator (SRC) under the MF induction, and a mutation in the PAS-b domain significantly contributes to the different induction effects by MF and JH III [9,38]. Compared with *Daphnia* or insect species, six of the eight JH binding sites [28] in the PAS-b domain are conserved, and the fifth residue is conserved in decapod crustaceans but is different from *Daphnia* or insects, while the seventh residue is conserved in crustacean species but is different from most of the insects. However, the fifth residue in decapod and *Daphnia* crustaceans is both polar and contains basic amino acid, while in insects, it is all non-polar and contains aliphatic amino acid, suggesting a similar property of crustaceans’ Mets.

To further analyze the binding properties between Met and JHs, in silico site-directed mutagenesis was conducted to predict the important role of contact residues when docking with MF. The interesting thing is that the wild type showed a similar interaction energy difference with MF or JH III; this may be because the residues Y312 and Y376 in Sp-Met, which are bound with the oxygen atom of epoxy group in JH III through the H-bond in the predicted model, are conserved in crustaceans and insects. Since *CYP15-like*, the gene catalyzing the transformation of MF to JH III, has also been found in crustaceans [39,40], the existence or not of JH III or other JHs in crustaceans will be further elucidated in the future. In addition, Y348F of Sp-Met increases the interaction energies with MF, which may be because of the change in cavity volume for MF binding. Another interesting thing is that all mutations increased the binding with JH III, and four of six mutations also increased the binding with MF. Since MF is also detected in insects, studies in *Drosophila* indicated that MF can act as a hormone itself or serve as the precursor of JHB3 to regulate the development process [41]. We propose that Mets of insects have evolved to have a higher affinity with both MF and JH III than crustaceans’ Mets.

The tissue distribution pattern of *Sp-Met* in female and male adult crabs was similar and a little different to the swimming crab and Chinese mitten crab [36,42], and this difference might mainly be due to the differences in the sampling stages in these species. The high expression in gonad tissues suggests that *Sp-Met* might participate in the MF regulation of reproduction processes of both females and males, while high expression in males’ ejaculatory duct and females’ seminal receptacle suggests that *Sp-Met* might also participate in the mating processes. This hypothesis can be supported by some evidence in other species. Significant variation in *Met* expression during the gonad development was found in both the swimming crab and Chinese mitten crab [36,42]. The knockdown of *Met* in the female desert locust resulted in a delayed display of copulation behavior with virgin males and an incapacity to oviposit during the time of the experimental setup [43]. Male insects may be able to coerce females into increasing the rate of oviposition either by directly transferring JH to females or by stimulating its synthesis using accessory gland proteins [44].

Compared with insects, the larva metamorphosis of crabs is prepared for growth but not for reproduction. However, MF can still inhibit larva metamorphosis in crustaceans [2,45]. The expression of both *Sp-Met* and *Sp-Kr-h1* was decreased and rarely detected during the Z5 to M metamorphosis and decreased before M to C1 metamorphosis but still had a certain amount of expression; this is consistent with the treatment effect of MF in our previous studies, in which MF could inhibit Z5 to M metamorphosis but only delay M to C1 metamorphosis [2]. Another interesting thing is that when it comes to the juvenile crab stage, the expression of *Sp-Met* seemed to increase from the first day until the last day of the molt cycle, suggesting that *Sp-Met* may also participate in molt regulation; this could be supported by some indirect evidence in other crustaceans [46,47], which deserves further exploration. Furthermore, the knockdown of *Sp-Met* on the first day of the Z4 stage caused some precocious metamorphosis phenotypes, including the degradation of the tissues in the spike at the carapace and the newborn chela bud, which normally appeared on the second day of the Z5 stage, suggesting that MF might be the crustacean JH preventing the larva from precocious metamorphosis; this can be supported by the increase in *Sp-Met* expression at the last day of Z4 stage. However, the knockdown of *Sp-Met* at Z4 did not cause a complete metamorphosis, which is different from insects [24]; this might be because the larva was not ready for metamorphosis [19,48]. Additionally, the knockdown of *Sp-Met* at the Z5 stage blocked the inhibitory metamorphosis effect of MF, indicating that this inhibitory effect was transduced by *Sp-Met*.

In addition, the expression of *Sp-Met* and *Sp-Kr-h1* varied significantly during full larval development, and these two genes have a very high correlation coefficient score, indicating that these two genes had a strong correlation. The knockdown of *Sp-Met* in Z4 significantly decreased the *Sp-Kr-h1* expression, while MF can rescue the reduction in *Sp-Kr-h1*, suggesting a conserved MF-Met→*Kr-h1* signal between the three crab species and insects [11,12], but it is different to that in the *Daphnia* species, in which the *Kr-h1* lost its responsiveness to JHs [10]. However, the knockdown of *Sp-Met* also significantly decreased the expression of two 20E signal genes, *Sp-EcR* and *Sp-E93*, and their expression increased when *Sp-Met* also increased, suggesting a positive relationship between *Sp-Met* and these two ecdysone signal genes. These are different from the antagonistic effects of JH and 20E in insects [4,23]. Since *EcR* was still responding to 20E in mud crabs [49], Met could prohibit or promote programmed cell death in the larval fat body in *Drosophila* under the existence of JH or not [50]. The crosstalk relationship between MF and 20E in crustaceans has become an interesting question deserving further exploration.

In conclusion, in this study, we characterized the gene and protein structure of Met from the mud crab, analyzed the binding properties between Met and JHs, examined the expression profiles in different tissues during larval development, and finally investigated the function of Met during the metamorphosis process using RNAi. The results indicate that Met could serve as an MF receptor to regulate larva metamorphosis, and the MF-Met→*Kr-h1* signal was conserved in mud crabs and insects.

## 4. Materials and Methods

### 4.1. Ethics Statement

All animal experiments in this study were conducted in compliance with the relevant national and international guidelines and were approved by the East China Sea Fisheries Research Institute.

### 4.2. Samples Collection

All samples were collected from the Zhejiang Ninghai research center of the East China Sea Fisheries Research Institute, Chinese Academy of Fishery Sciences. The adult mud crabs were collected in the culture pond in the center, and the larvae were collected from the nursery pond. For the mRNA tissue distribution analysis, twelve tissues, including ovary/testis (males), seminal receptacle/ejaculatory duct (males), stomach, midgut, hepatopancreas, heart, thoracic ganglia, hemolymph, muscle, antennae, antennule, and mandibular organ (MO), were dissected from nine adult female and male crabs. For the tiny tissues including MO, antennae and antennule, the same tissue from three individuals was pooled as one sample. Different stages of larval samples were collected at 8:00 a.m. every day during the full seed breeding process. From the Z2 to C2 stage, the first day of the specific stage was normalized by collecting the larvae on the last day of the previous stage in 22 L buckets. In total, 35 larval samples during larval development, including three in Zoea 1st (Z1) stage, three in Z2, four in Z3, five in Z4, five in Z5, one Z5 to M, nine in M, three in C1, and two in C2, were collected. Ten or three individuals were pooled as one sample for zoea and megalopa samples, respectively. All tissue or larvae samples had three parallels.

For RNA interference (RNAi) in Z4, the larvae on the last day of the Z3 stage, when a few larvae at the Z4 stage appeared but most were still at Z3 in the nursery pond, were collected and reared in the 22 L bucket; then, the larvae at the first day of Z4 stage were collected for the experiment in the morning of next day. The larvae on the last day of the Z5 stage were collected from the nursery pond for the Z5 experiment. The last day of the Z5 stage was indicated by the fact that a few megalopae appeared in the nursery pond and the near disappearance of the tissues in the spike of the carapace and newborn chela observed using micro-examination. All samples used for expression analysis were fixed in the RNA fixer and stored at −80 °C until the RNA extraction.

### 4.3. Sequence Analysis of Sp-Met

Met sequence was obtained from the genome and transcriptome database by searching for the function annotation results and further validated by using blast in the NR database. The coding region of Met was verified by PCR and Sanger sequencing with the primers of Met-F/R (Table 2). Gene structure was obtained using the Blat tool with the assembled genome [51] and the figure was drawn using the online tool GSDS 2.0 [52]. The open reading frame (ORF) and protein sequence of Met were predicted using the ORF finder (https://www.ncbi.nlm.nih.gov/orffinder, accessed on 12 March 2023), and conserved protein domains were predicted using the SMART [53].

### 4.4. Docking Analysis Between Met and MF

The 3D structure of Sp-Met was predicted using the AlphaFold2 [54]. Since the predicted result for the full sequence of Met was relatively low stable, we also predicted the PAS-PAC and its flank region structure of Sp-Met. Then, an in silico study was conducted to examine the binding properties between Sp-Met and MF or JH III by using the CB-DOCK2 [27]. Based on the alignment and docking results, six contact residues were identified when docking with MF, which were conserved in decapods but were different in insects as these residues were replaced by the corresponding residues. In addition, 3D structures of the six mutated sequences were also predicted, and docking between them and MF or JH III was conducted. The molecular graphics of the 3D structures were created with UCSF ChimeraX [55].

### 4.5. Expression Analysis

The total RNA of samples used for gene expression analysis was isolated by using the DNAaway RNA isolation kit (Sangon Biotech., Shanghai, China). The RNA concentration was determined by spectrophotometry, and the RNA integrity was determined by the visualization of clear bands of 18S and 28S ribosomal RNA on 1.0% agarose gel electrophoresis. A total of 0.2 µg of RNA was used for the synthesis of first-strand cDNA by reverse transcription reaction using ReverTra Ace^®^ qPCR RT Kit (TOYOBO, Osaka, Japan) and primer mix (oligo dT and random primers, provided by the kit) following the manufacturer’s instructions. The first-strand cDNAs from different samples were used separately as templates in 20 μL PCR reactions containing an SYBR green PCR master mix (TAKARA, Dalian, China) following the instructions. Each sample was measured in three duplicates. The reaction mixture was initially incubated for 15 min at 95 °C and then amplified over 40 cycles of denaturation at 95 °C for 15 s, followed by annealing and extension at 60 °C for 60 s. Specific amplification of each cDNA was verified by melting curve analysis, and DEPC water was used in place of the template in the negative control. Quantitative real-time polymerase chain reaction (qRT-PCR) was performed on a 7900 genetic analyser system (Applied Biosystems). Except for *Sp-Met*, the spatial expression of *Krüppel homolog 1* of the mud crab (Sp-*Kr-h1*) was also examined to examine the conservation of JH-Met→*Kr-h1* signal in crabs, and the *Sp-Kr-h1* sequence was obtained from our previously published genome data [51]. The *18S rRNA* fragment was used as an internal control due to its relatively stable expression [56]. All primers used in this study were summarized in Table 1. A relative standard curve was developed using 5-fold serial dilutions of cDNA. The standard curves were included in all runs to calibrate the quantitative data. The concentrations of cDNA in each sample were calculated from the standard curves.

### 4.6. Function Study of Sp-Met During Larval Metamorphosis

To further investigate whether *Sp-Met* could regulate larval metamorphosis, siRNA was used to knock down the expression of *Met*. The siRNA used for interference was synthesized by Sangon Biotech (Shanghai) Co. Ltd. (Shanghai, China), and the siRNA sequences were as follows: sense, GCCAAUGCUGACUCCAGAATT, and antisense, UUCUGGAGUCAGCAUUGGCTT. The siRNA targeting GFP was used as the negative control. The RNAi efficiency was examined using the hepatopancreas culture in vitro and Z4 stages larvae in vivo. For the in vitro experiment, the hepatopancreas tissues from three female crabs at the ovary developmental stage III (developing stage) were dissected and rinsed nine times with sterile saline. Then, the tissues were cut into small pieces of approximately 30 mg, and tissue fragment was placed into a well of the six-well culture plates containing 2 mL of high Glucose DMEM medium (Thermo Fisher Scientific, Inc., MA, USA). A total of 0.5 µg siRNA was added to the medium and the tissue fragments were collected at 8 h and 14 h for *Sp-Met* expression analysis. For the in vivo study, healthy Z4 stages larvae on the first day were transferred to the six-well cell culture plates with 2 mL sterile seawater in each cell, 0.5 µg siRNA was added at the start, and then after 12 h, 1 µmol/L (µM) MF was added in part of the iMet groups. The in vivo study was not only used for RNAi efficiency evaluation of *Sp-Met* but also for the examination of MF’s signal transduction. Therefore, the expression profiles of *Sp-Met*, *Sp-Kr-h1*, and two Ecd signal genes (*Sp-EcR* and *Sp-E93*, Supplementary File S1) were examined at 36 h and 96 h for the three groups, iGFP, iMet, and iMet + MF.

For the phenotype analysis, RNAi experiments were conducted in the 50 mL beakers with 20 mL sterile seawater, and 5 µg siRNA was added for the gene knockdown. Twenty larvae were added to each beaker and three parallels were set. Two experiments were conducted to examine the function of Met during metamorphosis. Since we have shown that MF could prohibit Z5 metamorphosis [2], the first experiment aimed to investigate whether this prohibitory role was transduced by *Met*. Four groups were set on the last day of the Z5 stage, including control, iGFP, 5 µM MF, and 5 µM MF + iMet groups; then, metamorphosis rates at different time points (24 h and 48 h) were collected and analyzed. The second experiment aimed to examine whether the knockdown of the *Met* could induce precocious metamorphosis. Two groups, including iGFP and iMet groups, were set on the first day of the Z4 stage, and after 24 h of treatment, the larvae were collected for phenotype analysis.

### 4.7. Statistical Analysis

All data obtained from the qRT-PCR analysis were log-transformed, and the mean and standard deviation (SD) of each sample were calculated. The gene expression and metamorphosis rates data were analyzed using the one-way ANOVA method, and the post hoc test was carried out using Tukey’s multiple comparison test using SPSS 22.0. The differences were considered significant at *p* < 0.05.

## Figures and Tables

**Table 1:** Docking analysis between Sp-Met and MF or JH III.

PPF of Sp-Met	Docking with MF		Docking with JH III	
	Vina Score (kcal/mol)	Cavity Volume (Å3)	Vina Score (kcal/mol)	Cavity Volume (Å3)
Wild type	−7.2	189	−7.2	189
T333A	−7.5	220	−7.8	220
L335Y	−7.6	633	−7.8	633
Y348F	−7.0	200	−7.5	228
S357V	−7.2	637	−7.7	637
Q361L	−7.7	574	−8.1	574
V374S	−7.3	205	−7.6	488

**Table 2:** Oligo nucleotide primers used in this study.

Name	Sequence (5′-3′)	Application
*Met*-F	CTCGTCCGAAGTTTGTTGCTG	PCR
*Met*-R	GATTGCCACAGAAAGGGCAGT	PCR
*Sp-Met*-RTF	GAACTGTGACTCGGATGGGG	Real time-PCR
*Sp-Met*-RTR	GACAACCCTCACGAAGCTGA	Real time-PCR
*Sp-Kr-h1*-RTF	GGGGACAAAAGGTGAGGCAT	Real time-PCR
*Sp-Kr-h1*-RTR	TTTGTCTCTCACAGCACGCA	Real time-PCR
*Sp-18S*-RTF	GGGGTTTGCAATTGTCTCCC	Real time-PCR
*Sp-18S*-RTR	GGTGTGTACAAAGGGCAGGG	Real time-PCR
*Sp-EcR*-RTF	AGCAGCCCGGTTCTATGATG	Real time-PCR
*Sp-EcR*-RTR	TCCCAAGCCAGCAAACTCAT	Real time-PCR
*Sp-E93*-RTF	CAAGAAGCTGGTGGAGCAGA	Real time-PCR
*Sp-E93*-RTR	TTCGCCTCCTCGTCAGAAAC	Real time-PCR

## Data Availability

The original contributions presented in this study are included in the article/Supplementary Material. Further inquiries can be directed to the corresponding author(s).

## Supplementary Materials

The following supporting information can be downloaded at https://www.mdpi.com/article/10.3390/ijms252312746/s1.
