# Supplementary figures and images for: Methoprene-Tolerant (Met) Acts as Methyl Farnesoate Receptor to Regulate Larva Metamorphosis in Mud Crab, Scylla paramamosain
Source: Int J Mol Sci. 2024 Nov 27;25(23):12746. doi: 10.3390/ijms252312746 (PMC11640826; doi:10.3390/ijms252312746)

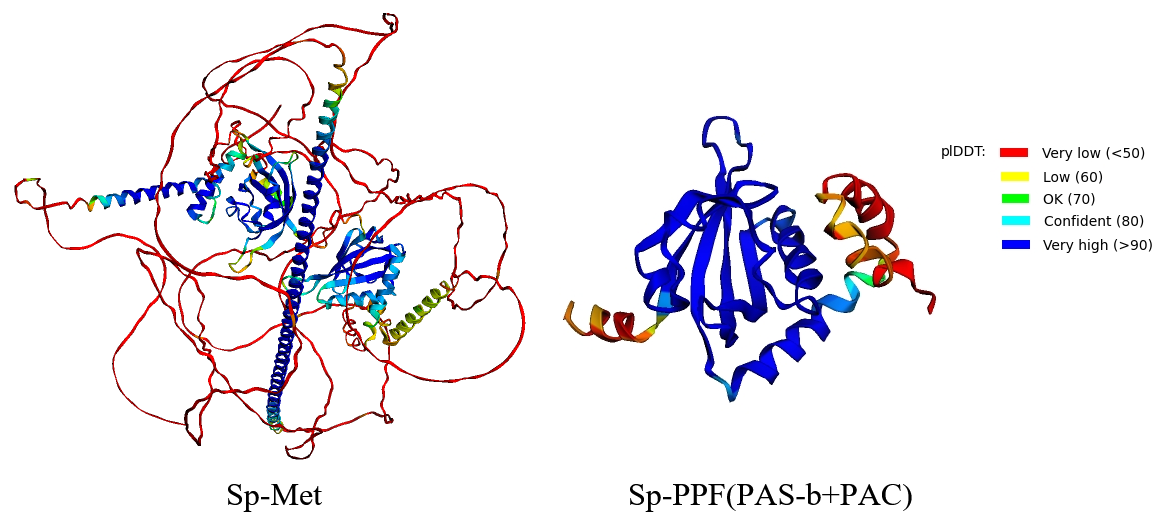

Supplement: Supplementary file 1 [file ijms-25-12746-s001.zip › Supplementary Figure S1.png]
